# Various Forms of Intermittent Diseases

**Published:** 1847-12

**Authors:** W. H. Martin

**Affiliations:** Rushville, Indiana


### ARTICLE III.

*Various Forms of Intermittent Diseases.* By Dr. W. H. Martin, of Rushville, Indiana.

The Physicians of our State, and probably of the whole Mississippi Valley, are more frequently called to prescribe for diseases of an Intermittent character than for any other form. That they all have their origin in a malarial cause, is generally conceded. That the same remedies are equally efficacious in all the multifarious cases presented to our care, our almost daily experience fully testifies. That there are cases, however, of this character, enveloped in such obscurity, and having such anomalous complications as to produce a conflict of opinions in the minds of many, particularly the juniors of the profession when called upon to treat them, cannot be doubted. If there be such, the relation of the following cases may not be uninteresting.

*Case* 1.—Mr. S. B., aged 54, of strong temperament and good constitution, arose early on the morning of the 5th of June, in good health, and after having taken his usual stroll on his farm, returned to the house and was attacked suddenly with pain. Being his family physician, he sent for me immediately. Upon enquiry I found that there had been no previous indisposition. The pain seemed to follow the course of the left ureter, and was very severe, of a persistent character, yet there were times when it was much more pungent and laucinating. The inclination to micturate was constant, and the urine passed off *guttatum.* No tenderness on pressure any where; extremities rather cool; pulse somewhat oppressed and about 65; tongue slightly coated.

Gave him sub. mur. hyd 20 grains,
pulv. opii 3 " and placed him in a warm hip bath, in which he remained about thirty minutes, much to his relief. He was then placed in bed, sinapisms put to his ankles, a dose of ol. ricini ordered in three hours, accompanied by forty drops of laudanum should there be a continuance of pain, and injections of a mild solution of mur. soda, if the oil did not operate in due time. *June 6th.*—Called to see my patient early, and found that the pain had entirely subsided in three hours after the time of attack on yesterday, and he had remained easy and felt entirely well until this morning, when there was a return of the pain accompanied by some chillness. He had taken, previous to my arrival this morning, 2 grains sulph. morphine, which I left during my visit yesterday, and was immediately placed in the warm bath. I remained with him about one hour, during which time the pain left him, and there came up slight fever. The medicine on yesterday produced copious alvine dejections of a good character.

Left fifteen grains quinine in five powders, to be commenced at 6 o'clock in the evening, and repeated every two hours until all were taken.

*June 7th.*—No return of pain. Cured.

*Case* 2.—Mr. B. T., aged 49. Habits irregular. Constitution pretty good. Was brought to our office in a waggon on the 10th of July, complaining of an excrutiating pain in the left shoulder, which he said made its attack on the evening previous. Upon examining the shoulder we found it somewhat swelled over the joint. Complained of soreness upon being handled, with entire inability to raise the arm. Would not submit to venesec.

Gave him a linament composed of spirits of ammonia, terebinth. and ol. lini, to be bathed freely over the joint.

Pulv. opii 2 grains,
Ipecac, 1 "
Nit. Pot. 5 " to be repeated every two and a half hours until relieved. Then to take an active purge of sulph. magnesia and senna.

Saw him again on the evening of the 12th. During the whole of yesterday, and until four o'clock this evening, the patient was entirely easy, but at this hour there was a return of all his former suffering. Put a warm hop poultice over the painful joint, and again placed him under the influence of anodynes, to be followed, after the subsidence of pain, with a dose of ol. ricini and spirits trerebinth. At six o'clock on the morning of the 14th, in anticipation of the evening paroxysm, he took three grains quinine, and repeated it every two hours until he had taken fifteen grains.

*July* 15.—Patient had no return of pain yesterday, and up to this time remains well.

*Case* 3.—On the 6th of May last, I was called in consultation by a neighboring physician to see a man who had suffered a short time previous with an attack of continued fever. but who had been discharged, some eight or nine days previously, by his physician as cured.

At the time of my being called in, he was afflicted with severe vomiting and purging, which I was informed had occurred on the two evenings previous, each paroxysm coming on about the same time, and leaving the patient easy but much prostrated. During these attacks the extremities were of an icy coldness. Upon the present occasion, we placed sinapisms on his ankles, wrists, and along the course of the spine. A flannel was wet with hot spirits of camphor, and laid on the epigastrum, and hot brandy toddy was occasionally administered. Under this treatment, the paroxysm was much shortened, and the supervening prostration lessened. He was then placed under full doses of quinine, from which time his convalescence was rapid. We have had a number of cases of this character, during the present season, every one of which yielded to the quinine treatment. Bleeding, blistering, sweating, and purging, with their multitude of inconveniences, in the treatment of a great majority of our summer and autumnal diseases, are fast becoming "things that were," much to the comfort and safety of our patients.

